# Compounding stress: A mixed-methods study on the psychological experience of miscarriage amid the COVID-19 pandemic

**DOI:** 10.1186/s12884-024-06610-z

**Published:** 2024-06-13

**Authors:** Madeline Fernandez-Pineda, Alison Swift, Christyn Dolbier, Kaitlin Guard Banasiewicz

**Affiliations:** 1 College of Nursing, Department of Nursing Science, East Carolina University, 2205 W 5th St, Greenville, NC 27834 USA; 2 College of Nursing, Department of Advanced Nursing Practice and Education, East Carolina University, 2205 W 5th St, Greenville, NC 27834 USA; 3 Department of Psychology, East Carolina University, Thomas Harriot College of Arts and Sciences, 104 Rawl Building, Greenville, NC 27858 USA; 4 College of Nursing, East Carolina University, 2205 W 5th St, Greenville, NC 27834 USA

**Keywords:** Miscarriage, COVID-19 pandemic, Pregnancy loss, Anxiety, Depression, PTSD, Psychological distress

## Abstract

**Background:**

Experiencing a miscarriage can have profound psychological implications, and the added strain of the COVID-19 pandemic may have compounded these effects. This study aimed to explore the psychological experiences, assess the levels of psychological distress (depression, anxiety, and post-traumatic stress disorder), and examine the relationships of personal significance of miscarriage and perceived stress with psychological distress of women in North Carolina who suffered a miscarriage of a desired pregnancy between March 30, 2020, and February 24, 2021, of the COVID-19 pandemic, at 14 to 31 months after the loss.

**Methods:**

We conducted a cross-sectional mixed-methods study using a convergent parallel design. A total of 71 participants from North Carolina completed the online survey and 18 completed in-depth interviews. The survey assessed demographics, mental health and reproductive history, personal significance of miscarriage, perceived stress, anxiety, depression, and PTSD. Interview questions asked about the psychological experience of the miscarriage and how the COVID-19 pandemic affected them and their experience.

**Results:**

Findings indicated moderate to severe levels of depression, anxiety, and PTSD, which persisted 14 to 31 months post-miscarriage. After conducting hierarchical binary logistic regressions, we found that perceived stress and prior trauma increased the odds of depression, perceived stress increased the odds of anxiety, and personal significance and prior trauma increased the odds of PTSD symptoms 14–31 months post-miscarriage. Notably, a subsequent successful childbirth emerged as a protective factor against depression, anxiety, and PTSD. Qualitative findings depicted emotions such as profound isolation, guilt, and grief. Women noted that additional pandemic-specific stressors exacerbated their distress. The categories identified via conventional content analysis fell under five broader thematic groups: mental health disorders, negative emotions/feelings, positive emotions/feelings, thoughts, and other experiences.

**Conclusions:**

Miscarriage during the COVID-19 pandemic intensified and added complexity to the psychological distress experienced by affected women. The study underscores the need for comprehensive mental health screenings, specialized support for vulnerable groups, and the necessity of trauma-informed care. Providers are strongly encouraged to adopt a multifaceted, individualized approach to patient care that is cognizant of the unique stressors introduced by the pandemic.

**Supplementary Information:**

The online version contains supplementary material available at 10.1186/s12884-024-06610-z.

## Introduction

In the United States (US), miscarriage, or the loss of a pregnancy before 20 weeks gestation, is the most common complication of pregnancy with an estimated 26% of all pregnancies and up to 10% of clinically reported pregnancies ending in miscarriage [1]. Miscarriage has been strongly associated with anxiety, depression, post-traumatic stress disorder (PTSD), and feelings of isolation due to poor social support and lack of loss acknowledgment that can last months to years after the loss [2, 3]. The psychological impact of miscarriage consists of trauma and bereavement for the child and the loss of imagining and planning for motherhood [4]. American women report more personal significance from the loss of the baby, and more feelings of devastation, isolation, and guilt compared to American men [5]. Women have expressed feelings of losing their identity tied to motherhood, coupled with sensations of personal failure, self-blame, guilt, and helplessness. Some immediately identify their pregnancy as a baby and prefer not to refer to it as a fetus or embryo [4].

Women who experience a miscarriage have increased rates of anxiety that can evolve into PTSD [6, 7]. Bereaved mothers tend to suffer clinically significant levels of anxiety with twice the odds of developing generalized anxiety disorder when compared to non-bereaved mothers in the first year after the loss [8]. In one study, 58 out of 338 women (17%) still reported moderate/severe anxiety at nine months follow-up [9]. Further, studies have shown that anxiety is more frequent and sustained than depression in women post-miscarriage [6]. However, oftentimes depression goes unrecognized despite it being reported to be four times higher in bereaved parents than in non-bereaved parents. Shaohua & Shorey [8] reported that one year after the loss, about 20% of women still struggled with the disabling symptoms of depression. High levels of stress have also been associated with an increased risk of miscarriage, while at the same time, experiencing miscarriage has been associated with post-traumatic stress [4, 9]. In one study, out of 336 women who experienced a miscarriage, 18% met the criteria for PTSD nine months after the loss [9].

During the times of the mandated stay-at-home and the recommended safer-at-home orders during the COVID-19 pandemic, there was an overall increase in mental health issues throughout the general US population [10, 11]. Several clinicians and experts raised concerns regarding the mental health of childbearing women during the COVID-19 pandemic. Childbearing women were coping with not only the status of their pregnancy but also with the prevention of or management of COVID-19 infection, hospitalizations and deaths of family members due to COVID-19 or other health issues, increased caregiving demands of children or family members, isolation from support systems and their community, job loss, financial hardships, etc [12, 13]. In North Carolina (NC), most counties (80%) are considered rural, with less than 250 residents per square mile [14], and the stay-at-home orders which spanned between March 30, 2020, and February 24, 2021 [15] added 11 months of further social isolation due to spikes of COVID-19 variants on top of overwhelmed healthcare systems. All these compounding factors may have considerably aggravated the psychological distress of women experiencing a miscarriage in NC. Although miscarriage is a common occurrence, there are few studies conducted in the US that focus on the psychological impact and even fewer that assess the psychological impact of miscarriage amidst a global pandemic.

Given the profound psychological consequences of miscarriage, coupled with the heightened stress of the COVID-19 pandemic, there exists a gap in understanding the psychological experiences of women who suffered a miscarriage during this period. Especially in states like North Carolina where unique factors like the predominance of rural areas may exacerbate feelings of isolation, it becomes critical to delve into these experiences to offer better support and care. It is within this context that this study aims to (1) explore the psychological experiences, (2) assess the levels of psychological distress (depression, anxiety, and PTSD), and (3) examine the relationships of personal significance of miscarriage and perceived stress with psychological distress (depression, anxiety, PTSD) of women in North Carolina who suffered a miscarriage of a desired pregnancy between March 30, 2020 and February 24, 2021 of the COVID-19 pandemic, at 14 to 31 months after the loss.

## Method

### Design

We conducted a cross-sectional mixed-methods study using a convergent parallel design to address the aims. This design allows for quantitative and qualitative data collection to occur independently and simultaneously with results converged after separate analysis [16]. The University and Medical Center Institutional Review Board at East Carolina University approved the study prior to initiation.

### Participants and recruitment

Inclusion criteria for this study were: women (as identified by biological sex); who experienced a miscarriage (< 20 weeks gestation) of a desired pregnancy between March 30, 2020, and February 24, 2021; resided in North Carolina; were 18 years or older; and able to speak and read in English or Spanish. The exclusion of stillbirths, defined as pregnancy loss after 20 weeks, was due to differences in medical and psychological management compared to miscarriages. Miscarriages usually occur in outpatient or emergency settings with less follow-up support, while stillbirths often require more intensive and comprehensive medical support [17, 18]. Additionally, the inability to grieve a tangible baby after a miscarriage impacts people’s experiences differently than stillbirths, where formal mourning is more common [18, 19].

Using a power analysis for binary logistic regression, assuming power = 0.80, α = 0.05, and a medium odds ratio of 2.48 [20], the estimated sample size needed was 71. Participants were recruited between May and September 2022 through convenience and snowball sampling. A study flyer was created containing study information, Primary Investigator (PI) contact information, and the link to the Research Electronic Data Capture (REDCap) survey, in English and Spanish. The flyer was distributed through a large public university faculty/staff mailing listserv, a newsletter at a major public hospital, and posted at one primary care and two Obstetrics and Gynecology (OB/GYN) clinics in eastern NC and 22 health department clinics across NC.

A social media script containing the study information, PI contact information, and link to the survey, in English and Spanish, was also sent to the administrators of Facebook groups across NC. Facebook is the most widely used social media platform in the US, with 77% of women and 70% of US adults between the ages of 18–49 considering themselves users [21]. The shareable study flyer was posted to 437 Facebook pages, which included NC miscarriage/pregnancy loss support group pages, mom groups, health department pages, and community groups.

We also utilized a nested sampling approach and invited participants who provided their email addresses in the survey for an in-depth interview [22]. According to Hennink et al. [23], a sample of 16 to 24 in-depth interviews is needed to fully understand the conceptual codes identified during analysis. Thus, the research team invited participants for this portion of the study until a total of 16 to 24 participants completed an in-depth interview (in English or Spanish depending on their preference).

### Procedures

The second author translated several of the English study documents into Spanish using electronic translation software. Then the bilingual PI reviewed them for accuracy. Electronic informed consent was obtained for both the survey and interview, followed by the eligibility screening questions. Eligible participants could advance to the survey.

The in-depth interviews were offered in person at a location of the participant’s choosing, or via Microsoft Teams audio-conferencing service. Interviews were recorded using the audio recording integrated into Microsoft Teams. Precautions to avoid the spread of COVID-19 were taken for in-person interviews by following the most current Centers for Disease Control and Prevention recommendations at the time of data collection. During each interview, the PI reviewed the participant’s rights and answered study-related questions. The PI and another study team member then moderated the conversation using a semi-structured interview guide and took field notes [24]. The interview questions were developed based on, existing practice and research and included topics about women’s miscarriage experience, including their psychological experience and effects of the COVID-19 pandemic (see supplementary file). Interviews took between 42 and 112 min.

Additionally, questions on satisfaction with provider care, coping strategies, social support, future pregnancies, and preferences for emotional support interventions were also included in both the survey and interviews but are not addressed by the research aims of this paper. Contact information regarding local and national support groups, a suicide hotline phone number, and local counseling services that participants could access if needed was provided at the end of the survey and sent via email to participants after the interviews. Participants were compensated with a $50 Clincard for their time upon completion of the interviews.

### Assessments

#### Demographic, reproductive, mental health, and COVID-19 stress

Demographic questions included age, marital status, race, ethnicity, annual income, education level, employment status, geographic residence, and type of health insurance coverage. Reproductive questions included reproductive history, number of miscarriages and timing of each, weeks of gestation at the time of loss, miscarriage location (home, hospital, and/or other), miscarriage management, and history of infertility treatment. Yes/no mental health questions asked whether participants had any mental health diagnosis and any other traumatic event in their life before the pandemic miscarriage, not including prior pregnancy losses. We also asked participants how much the COVID-19 pandemic caused stress to their lives while they had the miscarriage, using a 5-point Likert ranging from 1 (Not at all) to 5 (Extremely; see supplementary file).

#### Personal significance of miscarriage

Appraisal of the meaning of the experience of miscarriage was measured using the Revised Impact of Miscarriage Scale [25] (RIMS). The RIMS is a 16-item measure using a 4-point Likert scale ranging from 1 (Definitely true for me) to 4 (Definitely not true for me) and includes three subscales. The isolation/guilt subscale consists of 6 items (sample item: “I feel much alone in my loss”). The loss of baby subscale consists of 5 items (sample item: “I feel there will always be a place in my heart for the miscarried baby”). The devastating event subscale consists of 5 items (sample item: “My miscarriage was a horrendous, devastating event”). Participants were asked to think about “the time you experienced the miscarriage(s) during the COVID-19 pandemic” when responding to these items. Items are reverse scored prior to summing items for subscale and total scores. Higher scores indicate more meaning and personal significance. The RIMS was validated in US women experiencing miscarriage, with internal consistency estimates ranging from 0.65 to 0.79 [5, 25]. Cronbach’s alpha for the current study was 0.92 for the total scale and 0.78–0.85 for the subscales.

#### Perceived stress

Perceived stress was measured using Perceived Stress Scale [26] (PSS-4). The PSS-4 is a 4-item measure using a 5-point Likert scale ranging from 0 (Never) to 4 (Very often). Participants were asked to think about “the stress you may have felt after the miscarriage(s) you experienced during the COVID-19 pandemic” when responding to these items. Two items are reverse scored prior to summing all items. Scores range from 0 to 16 with higher scores indicating more perceived stress. The PSS-4 is a reliable tool to measure stress in pregnant women [27], and has demonstrated adequate internal consistency, with a Cronbach’s alpha = 0.79 in English [27] and 0.76 in Spanish [28]. Cronbach’s alpha for the current study was 0.78.

#### Anxiety

Anxiety was measured with the Generalized Anxiety Disorder Scale [29] (GAD-7). This 7-item measure uses a 4-point Likert scale ranging from 0 (Not at all) to 3 (Nearly every day). Participants were asked to think about “the time you experienced the miscarriage(s) during the COVID-19 pandemic” when responding to these items. Items are summed for a total score. Scores are interpreted as follows: minimal (0–4), mild (5–9), moderate (10–14), and severe (15–21), with scores of 10 or above indicating a likely clinical diagnosis [29]. The GAD-7 has been previously used in women experiencing miscarriage [30]. Cronbach’s alpha for the current study was 0.93.

#### Depression

Depression was measured using the Patient Health Questionnaire [31] (PHQ-8). This 8-item measure uses a 4-point Likert scale ranging from 0 (Not at all) to 3 (Nearly every day). Participants were asked to think about “the time you experienced the miscarriage(s) during the COVID-19 pandemic” when responding to these items. Items are summed for a total score. Scores are interpreted as follows: minimal (0–4), mild (5–9), moderate (10–14), moderately severe (15–19), and severe depression (20–24), with scores of 10 or above indicating a likely clinical diagnosis [31]. The PHQ-8 is a well-validated tool in English and Spanish [31, 32] and has been used in the study population [33]. Cronbach’s alpha for the current study was 0.93.

#### PTSD

PTSD was measured using the Primary Care for PTSD screen for DSM-5 scale (PC-PTSD-5), which consists of five yes/no items to assess PTSD symptoms over a one-month period [34]. In the current study, participants were asked to think about “the time you experienced the miscarriage(s) during the COVID-19 pandemic” when responding to items. Three or more “yes” responses indicate probable PTSD [34]. A recent study on pregnancy outcomes of childbearing women indicated a strong Cronbach’s alpha of 0.89 [35]. Cronbach’s alpha for the current study was 0.68.

### Data Analysis

#### Survey

The quantitative data were analyzed using SPSS version 28. Descriptive statistics were calculated to describe the demographic, reproductive, and mental health characteristics of the sample and subsample of participants who completed interviews. To address Aim 2, descriptive statistics for the key study variables for the total sample were calculated. We conducted three separate hierarchical binary logistic regressions to address Aim 3. Each of the regressions included a dichotomous criterion variable of psychological distress based on meeting the clinical cutoffs for the PHQ-8 for depression, GAD-7 for anxiety, and PC-PTSD-5 for PTSD. Block 1 predictors included control variables of mental health diagnosis prior to pandemic pregnancy, trauma other than pregnancy loss prior to pandemic pregnancy, and having a baby since pandemic miscarriage or currently being pregnant. Block 2 predictors included personal significance of miscarriage and perceived stress related to miscarriage.

#### In-depth interviews

In-depth interviews addressed Aim 1 (explore the psychological experiences of miscarriage). Microsoft Teams audio recordings were transcribed by Microsoft Teams in both English and Spanish. The PI and a study team member reviewed the transcripts for completeness. There was only one Spanish transcript, and it was translated into English using electronic translation software then verified by the bilingual PI before analysis.

The research team used NVivo12 to analyze the transcripts and organize findings, creating a clear audit trail and codebook. The research team followed six steps of thematic conventional content analysis. The PI and the second author independently coded three identical transcripts to generate initial codes [36, 37]. After discussing their findings, they developed a unified initial codebook. They then independently applied this codebook to analyze another set of two identical transcripts, and then met again to compare codes. Codes were reviewed, modified, and further developed to ensure they were relevant and did not overlap, leading to a more developed codebook. At this point, the two coders each took about half of the remaining transcripts and independently analyzed them further collapsing codes into categories [36]. In the end, they met again with the third author to come to a consensus on defining and naming the overarching themes. Method triangulation, which includes analysis of field notes, was also conducted to ensure the accuracy of the qualitative findings [38].

#### Data integration

We conducted an interpretive integration of the results using a joint display table to compare the qualitative findings with the quantitative data and develop an overall interpretation [16]. We identified common concepts across the datasets and developed a joint display table to array the results side by side, organized by these concepts. This joint display facilitated a direct comparison of the qualitative themes and categories with the corresponding surveys and survey items, enabling us to determine whether the data supported each other [16]. We indicated convergence when both qualitative and quantitative results aligned. Where the quantitative data did not capture or address some qualitative findings, we noted a lack of convergence. These qualitative findings that did not converge were considered an expansion of our understanding of the studied phenomenon, providing new insights for further exploration. In the Results section below, we first present the quantitative data, followed by the qualitative data, and then the integrated findings.

## Results

### Sample

A total of 71 participants met the inclusion criteria and completed the online survey. Out of these, 41 participants provided their email addresses, indicating their willingness to be contacted for an interview. Of those, interviews were completed with 18 participants. Initially, we tried to invite participants with purpose to increase racial/ethnic diversity but when we received insufficient responses and hadn’t yet reached data saturation, we altered our approach and contacted participants sequentially, starting with those who had first completed the survey. We achieved data saturation with the first 16 interviews. However, we chose to conduct two additional interviews with women from underrepresented racial/ethnic populations who had responded to our invitation to increase representation.

Tables 1 and 2 present the sociodemographic, reproductive, and mental health characteristics of the total sample (*N* = 71), and the sub-sample who were interviewed (*n* = 18). For the total sample, age ranged from 18 to 45 (*M* = 32.4, *SD* = 5.8), with most being between 30 and 39 (52.8%). Most of the women were White (83.1%), non-Hispanic/non-Latina (93.0%), married (78.9%), had at least a baccalaureate degree (52.1%), worked full time (54.9%), had private health insurance (73.2%), had annual household incomes of > $50,0000 (69.0%), and lived in suburban or urban areas (57.8%; Table 1). Regarding reproductive history, most participants had been pregnant three or more times (67.6%, range 1–10, *M* = 3.6, *SD* = 1.9), had at least one child (80.3%, range 0–5, *M* = 1.4, *SD* = 1.1), and had experienced one pregnancy loss (54.9%, range 1–5, *M* = 1.6, *SD* = 0.9). Most participants indicated no mental health diagnoses (59.2%) and no trauma other than pregnancy loss (53.5%) prior to pandemic pregnancy. Most participants reported one pregnancy loss during this period (73.2%, range 1–4, *M* = 1.3, *SD* = 0.6), occurring in the first trimester (94.7%), at home (78.9%), and via expectant management (63.4%). Since the designated pandemic period, most had since had a baby or were pregnant at the time of the study (60.6%; Table 2).

**Table 1:** Categorical sociodemographic characteristics of study participants

Characteristic	Total sample (*N* = 71)		Interview sub-sample (*n* = 18)	
	*n*	%	*n*	%
Ethnicity				
Non-Hispanic/non-Latina	66	93.0	15	83.3
Hispanic/Latina	5	7.0	3	16.7
Race				
White	59	83.1	14	77.8
Black	6	8.5	2	11.1
American Indian/Alaska Native	3	4.2	0	0
Native Hawaiian/other Pacific Islander	1	1.4	1	5.6
Multiracial or other	2	2.8	1	5.6
Marital status				
Married	56	78.9	15	83.3
Committed relationship	8	11.3	3	16.7
Never married	5	7.0	0	0
Divorced/separated	2	2.8	0	0
Education				
High school/GED	9	12.7	1	5.6
Technical school	12	16.9	2	11.1
Some college	13	18.3	2	11.1
Baccalaureate degree	18	25.4	8	44.4
Some post-baccalaureate or graduate degree	19	26.8	5	27.8
Employment				
Full time	39	54.9	10	55.6
Part time	13	18.3	3	16.7
Not employed	19	26.8	5	27.8
Annual household income				
≤ $25,000	7	9.9	1	5.6
$26,000–50,000	15	21.1	3	16.7
$51,000–75,000	15	21.1	3	16.7
$76,000–100,000	8	11.3	3	16.7
$101,00-150,000	15	21.1	6	33.3
> $151,000	11	15.5	2	11.1
Residence				
Rural	30	42.3	5	27.8
Suburban	30	42.3	1	5.6
Urban	11	15.5	12	66.7
Insurance				
Private	52	73.2	16	88.9
Medicare/Medicaid	14	19.7	1	5.6
Other	2	2.8	0	0
None	3	4.2	1	5.6

**Table 2:** Reproductive and mental health characteristics of study participants

Characteristic	Total sample (*N* = 71)		Interview sub-sample (*n* = 18)	
	*n*	%	*n*	%
# total pregnancies				
1	5	7.0	1	5.6
2	18	25.4	4	22.2
3	20	28.2	4	22.2
> 3	18	25.4	9	50.0
Number of children				
0	14	19.7	1	5.6
1	26	36.6	8	44.4
2	23	32.4	9	50.0
>2	8	11.2	0	0
# total pregnancy losses				
1	39	54.9	7	38.9
2	26	36.6	9	50.0
>2	6	8.5	2	11.1
Mental health diagnosis before pandemic pregnancy	29	40.8	9	50.0
Other trauma before pandemic pregnancy	33	46.5	11	61.1
OB/GYN diagnoses interfering with fertility	13	18.3	3	16.7
Pandemic pregnancy result of infertility treatment	11	15.5	2	11.1
# pandemic miscarriages (3/30/20 − 2/24/21)				
1	52	73.2	12	66.7
2	15	21.1	5	27.8
>2	4	5.6	1	5.6
Pandemic miscarriage weeks gestation				
0–13 weeks	90	94.7	25	100
14–20 weeks	5	5.3	0	0
Pandemic miscarriage location				
Home	56	78.9	14	77.8
Hospital	25	35.2	8	44.4
Other	8	11.3	2	11.1
Pandemic miscarriage management				
Medical	14	19.7	3	16.7
Surgical	25	35.2	8	44.4
Expectant	45	63.4	10	55.6
Reproductive history since 2-24-21				
Had baby	34	47.9	11	61.1
Currently pregnant	9	12.7	2	11.1
No pregnancies	28	39.4	5	27.8

Overall, the subsample had similar demographic and reproductive characteristics as the total sample, with a few exceptions. Compared to the total sample, the subsample had a narrower age range (27–43 years), all participants were married or in a committed relationship, and greater percentages were Hispanic/Latina, had earned Baccalaureate degrees, had more annual incomes in the $101,00-150,000 range, lived in urban regions, and had private health insurance. Regarding reproductive characteristics, the subsample participants had more than three pregnancies, at least one or two children, at least two pregnancy losses, had a mental health diagnosis and other trauma before the pandemic pregnancy, and had a baby since the pandemic period.

### Quantitative results

#### Descriptive statistics

Descriptive statistics for the key study variables for the total sample are reported in Table 3. On average, participants reported moderately high levels of personal significance of the pandemic miscarriage(s) (RIMS) overall and on its three subscales (see supplementary file for additional descriptive statistics for the RIMS). Participants reported moderate levels of perceived stress after the pandemic miscarriage(s) and moderately high levels of stress due to the COVID-19 pandemic at the time of the miscarriage(s). Participant levels of psychological symptoms after their pandemic miscarriage included moderate to severe anxiety (47.9%), moderate to severe depression (38.0%), and likelihood of PTSD (64.8%).

**Table 3:** Descriptive statistics of study variables

Variable	*M*	*SD*	Range	*n*	%
Total impact of miscarriage(s)	50.13	9.55	21–64		
Devastating event	16.65	3.11	6–20		
Isolated guilt	17.01	4.25	6–24		
Loss of baby	16.27	3.33	7–20		
Perceived stress related to miscarriage(s)	9.00	3.41	2–16		
COVID stress at time of miscarriage(s)	3.76	1.22	1–5		
Anxiety symptoms related to miscarriage(s)	9.32	5.84	0–21		
Minimal - mild				37	52.1
Moderate - severe				34	47.9
Depression symptoms related to miscarriage(s)	8.74	6.93	0–24		
Minimal - mild				44	62.0
Moderate - severe				21	38.0
PTSD symptoms related to miscarriage(s)	3.07	1.58	0–5		
Unlikely				25	35.2
Likely				48	64.8

### Inferential statistics

Table 4 presents the logistic regression results that examine relationships of personal significance of miscarriage and perceived stress related to miscarriage with psychological symptoms (depression, anxiety, PTSD) 14 to 31 months after miscarriage. For all three regressions, block 1 with the control variables (prior trauma, mental health history, subsequent baby/pregnancy) was significant, and block 2, adding personal significance and perceived stress, showed a significantly improved model fit. The full model including personal significance, perceived stress, and the control variables was significant for psychological symptoms of depression (*C*^*2*^ = 37.31, *p* < .001), anxiety (*C*^*2*^ = 33.84, *p* < .001), and PTSD (*C*^*2*^ = 29.18, *p* < .001). Significant predictors of meeting the clinical cut-off for depression included perceived stress (*B* = 0.47), prior trauma (*B* = 2.20), and having had a baby since the miscarriage (*B* = − 1.71). Participants were more likely to have a positive screen for depression if they had higher perceived stress (OR 1.60, 95% CI 1.22 to 2.11, *p* = .001) and prior trauma (OR 9.03, 95% CI 2.10 to 38.76, *p* = .003); however, they were less likely to have a positive screen for depression if they had a baby since the miscarriage (OR 0.18, 95% CI 0.04 to 0.80, *p* = .024). Significant predictors of meeting the clinical cut-off for anxiety included perceived stress (*B* = 0.37) and having had a baby since miscarriage *B* = − 2.00). Participants were more likely to screen positive for anxiety if they had higher perceived stress (OR 1.45, 95% CI 1.16 to 1.83, *p* = .001), and participants who had a baby since the miscarriage had decreased odds of a positive screen for anxiety (OR 0.14, 95% CI 0.03 to 0.56, *p* = .006). Significant predictors of meeting the clinical cut-off for PTSD included personal significance (*B* = 0.11), prior trauma (*B* = 1.91), and having had a baby since the miscarriage (*B* = − 2.09). Participants were more likely to have a positive screen for PTSD if they had higher personal significance (OR 1.12 95% CI 1.03 to 1.21, *p* = .006) and prior trauma (OR 6.74, 95% CI 1.59 to 28.59, *p* = .01), and they were less likely to screen positive for PTSD if they had a baby since the miscarriage (OR 0.12, 95% CI 0.03 to 0.56, *p* = .007).

**Table 4:** Logistic regression results examining personal significance of miscarriage and perceived stress with depression, anxiety, & PTSD

	*X* ^*2*^	*df*	*p*	*R* ^2^_*N*_	*B*	*SE*	*Wald*	*p*	*OR*	95% CI
Depression										
Block 1	19.02	4	< 0.001	0.32						
Block 2	18.29	2	< 0.001							
Full model	37.31	6	< 0.001	0.56						
Prior trauma					2.20	0.74	8.75	0.003	9.03	2.10, 38.76
Mental health history					-0.87	0.73	1.42	0.234	0.42	0.10, 1.76
Baby since miscarriage					-1.71	0.76	5.07	0.024	0.18	0.04, 0.80
Currently pregnant					-1.64	1.09	2.25	0.133	0.19	0.02, 1.65
Perceived stress related to miscarriage					0.47	0.14	11.44	0.001	1.60	1.22, 2.11
Personal significance of miscarriage					-0.01	0.05	0.01	0.940	1.00	0.91, 1.09
Anxiety										
Block 1	19.31	4	< 0.001	0.32						
Block 2	14.53	2	< 0.001							
Full model	33.84	6	< 0.001	0.51						
Prior trauma					1.19	0.67	3.18	0.074	3.28	0.89, 12.06
Mental health history					0.72	0.64	1.26	0.262	2.06	0.58, 7.25
Baby since miscarriage					-2.00	0.72	7.62	0.006	0.14	0.03, 0.56
Currently pregnant					0.28	1.02	0.08	0.782	1.32	0.18, 9.68
Perceived stress related to miscarriage					0.37	0.12	10.33	0.001	1.45	1.16, 1.83
Personal significance of miscarriage					0.01	0.04	0.01	0.994	1.00	0.93, 1.08
PTSD										
Block 1	19.27	4	< 0.001	0.33						
Block 2	9.91	2	0.007							
Full model	29.18	6	< 0.001	0.46						
Prior trauma					1.91	0.74	6.69	0.010	6.74	1.59, 28.59
Mental health history					-1.07	0.74	2.09	0.148	0.34	0.08, 1.46
Baby since miscarriage					-2.09	0.77	7.38	0.007	0.12	0.03, 0.56
Currently pregnant					-0.51	1.00	0.26	0.611	0.60	0.09, 4.25
Perceived stress related to miscarriage					0.03	0.10	0.07	0.798	1.03	0.84, 1.25
Personal significance of miscarriage					0.11	0.04	7.68	0.006	1.12	1.03, 1.21

*Note* Covariates: assigned no prior trauma = 0, prior trauma = 1; no mental health diagnosis history = 0, mental health diagnosis history = 1; did not have baby or pregnancy since loss = 0, had baby since loss = 1, currently pregnant = 2; OR = odds ratio, 95% CI = lower and upper bound of 95% confidence interval

### Qualitative results

The qualitative data from this study were classified into a series of themes and categories that build on the quantitative findings presented above and further elucidate the psychological experiences of participants following their miscarriage amid the COVID-19 pandemic (aim one). The categories fell under five broader thematic groups: *mental health disorders*, *negative emotions/feelings*, *positive emotions/feelings*, *thoughts*, and lastly *other experiences* which consisted of categories that were part of participants’ overall psychological experiences but did not fit under the mental disorders, emotional, and cognitive themes. It is crucial to understand that the categories nestled within these thematic groups are multifaceted constructs. They often embody a mix of emotions, feelings, and thoughts, reflecting the complex interplay of the psychological experiences following a miscarriage. Table 5 specifies the overarching theme each category belongs to, each category’s definition, direct participant quotes to exemplify each category, and outlines the specific timeline during which participants reported these emotions/feelings or thoughts.

**Table 5:** Miscarriage psychological experience during the COVID-19 pandemic

Timeline of Events	Themes & Categories	Description	Participant Quote
**Mental Health Disorders**			
Before Loss Confirmed, During or Immediately After Loss Confirmed, Short time (Days to first few months), 14 to 31 Months After Loss Confirmed	Anxiety	Data was categorized as *anxiety* when participants stated being diagnosed with anxiety after the miscarriage and how they felt the anxiety was affecting their health and their life.	*“My anxiety was crippling. Literally crippling like I I I really like couldn’t function and my husband really needed to take care of my son who had just turned 3 at the time, so it was extremely isolating.”* (ID: 101, 33 y/o, non-Hispanic/non-Latina White)
Before Loss Confirmed, Short time (Days to first few months), 14 to 31 Months After Loss Confirmed	Depression	Data was categorized as *depression* when participants stated being diagnosed with depression after the miscarriage, if they mentioned symptoms indicative of depression, or if they stated they felt depressed.	*“I think it’s really hard anyway to go in and into admit that you’re in a in that kind of mental space at the time. You know, like you’re not sleeping at night. You’re not eating your stomach upset. You’re shaking. You’re having intrusive thoughts. You can’t concentrate on work. You don’t wanna get out of bed, you know, even to talk to, to someone about any of those things.”* (ID: 101, 33 y/o, non-Hispanic/non-Latina White) *“So that was the first miscarriage I had and then a couple months later I had another miscarriage and after that one I became really depressed and I believe I just felt like my body had failed at what it was supposed to be doing, and I felt like I didn’t have purpose anymore, and I pretty much became suicidal for a little while after that.”* (ID: 178, 28 y/o, Hispanic/Latina White)
Before Loss Confirmed, Short time (Days to first few months), 14 to 31 Months After Loss Confirmed	PTSD	Data was categorized as *PTSD* when participants stated being diagnosed with PTSD after the miscarriage, if they mentioned symptoms indicative of PTSD, or if they stated they felt they were experiencing PTSD.	*“Going into the ultrasound room, uh, I felt like I was gonna have a panic attack. Um. Like I couldn’t breathe or that was holding my breath until I heard the heartbeat. I feel like it was like PTSD honestly, it it it was just being in that room it had been nothing but bad news. And it was almost like my I was trained to think you know I’m gonna be getting more bad news.”* (ID: 38, 33 y/o, non-Hispanic/non-Latina White, subsequent pregnancy)
**Negative Emotions/Feelings**			
Short time (Days to first few months)	Anger	Data was categorized as *anger* when participants expressed feelings of anger, or the strong displeasing emotion when something unfair or unkind happened to them.	*“So once we got past that first three weeks or so, most days I was OK, most days I could proceed on as normal as possible, but then there was days where I was just angry. Like why did this happen to me? There were days where, like I would become very upset because like what, again, what does pregnancy and fertility look like for me in the future?”* (ID: 69, 24 y/o, non-Hispanic/non-Latina White)
During or Immediately After Loss Confirmed, Short time (Days to first few months), 14 to 31 Months After Loss Confirmed	Resentment	Data was categorized as *resentment* when participants indicated they felt displeasure and bitterness due to experiencing an unjust situation.	*“Looking back, like I was very angry, like they never even wanted children. They got pregnant because her best friend was getting pregnant. They had a healthy baby, like I’ve lost my baby.”* (ID: 69, 24 y/o, non-Hispanic/non-Latina White) *“I hate to say I resented my husband, but he would be over there taking a nap, and I’m just like, I’m over here losing a baby and you’re over there just taking a nap.”* (ID: 30, 28 y/o, non-Hispanic/non-Latina White)
During or Immediately After Loss Confirmed, Short time (Days to first few months)	Helpless	Data was categorized as *helpless* when participants indicated there was nothing anyone could do to prevent the miscarriage or that there aren’t always answers to why it happens.	*“So, at the after my D&C for my follow up for my D&C, when I asked her, you know, what can I do to make my body not reject pregnancy and she said ‘well, you’re doing everything right, you know you’re taking your vitamins, you’re eating like you’re supposed to you know you’re doing’. She said, ‘you’re healthy’, she said, ‘you’re doing all that you can do’ so, you know, it’s just hard in the in the sense in that moment when you think that there’s more that you can do and when really there’s not so.”* (ID: 30, 28 y/o, non-Hispanic/non-Latina White)
Before Loss Confirmed, During or Immediately After Loss Confirmed, Short time (Days to first few months), 14 to 31 Months After Loss Confirmed	Trauma	Data was categorized as *trauma* when participants used the word themselves to describe their experience.	*“…uh I did take it to the office to the fact that I passed to for them to send it off to get test run on it, but, and that was traumatizing, too, like having to collect that out of the toilet…”* (ID: 38, 33 y/o, non-Hispanic/non-Latina White) *“No, I don’t think so, no, because I’m pretty sure, if I remember correctly, I think I passed in our toilet before I left like I’m pretty sure I looked down and there was a massive like clot which was even worse traumatic like did I just flush our baby down the toilet, it never having had a miscarriage before, right? And I was like, does that what I just did?”* (ID: 63, 38 y/o, non-Hispanic/non-Latina White)
During or Immediately After Loss Confirmed, Short time (Days to first few months)	Lonely	Data was categorized as *lonely* when participants indicated feeling like they were the only ones going through this loss, alone in their thoughts, alone in their experience and no one could understand what they were going through.	*“…but emotionally I was so distraught and broken and I felt like no one understood at all, like they came close to understanding, and so I felt absolutely alone even though I knew a lot of other women go through the same thing, it’s different when you’re going through it yourself.”* (ID:178, 28 y/o, Hispanic/Latina White)
During or Immediately After Loss Confirmed, Short time (Days to first few months)	Isolated	Data was categorized as *isolated* when participants indicated being left alone either at the hospital or at home while suffering the loss and how that affected their experience.	*“…I was there almost 6 h and yet the entire time just sitting there and I think the doctor came in and spoke with me three times, I went and had a ultrasound done just to kind of confirm everything, I went through blood work and I spoke with the nurse staff I had maybe a grand total of four times in that six hour time span, so it was a lot of just kind of being stuck in a room by yourself alone and feeling detached from the world, if that makes sense.”* (ID: 109, 27 y/o, non-Hispanic/non-Latina White)
During or Immediately After Loss Confirmed, Short time (Days to first few months), 14 to 31 Months After Loss Confirmed	Shame	Data was categorized as *shame* when participants indicated they felt embarrassed because of their actions, their situation, or their thoughts.	*“…so I actually hid my first miscarriage from people I didn’t even say nothing because I was kind of, like, embarrassed, another strange, but I’m like, Oh my goodness, and then when the second one came, I wasn’t gonna tell anybody,… I remember apologizing over and over again like sorry for making a mess in the hospital because, like, the blood was just so much as, like so many puddles of blood, and, like, I drip blood from going in and going to the bed and it was just embarrassing for me, painful and embarrassing.”* (ID: 102, 28 y/o, non-Hispanic/non-Latina Black)
Before Loss Confirmed, During or Immediately After Loss Confirmed, Short time (Days to first few months), 14 to 31 Months After Loss Confirmed	Guilt	Data was categorized as *guilt* when participants described feeling as if they themself did something wrong to cause the miscarriage.	*“…of why me? What did my body do wrong? How did I mess up? Why did like? Because I blamed myself, but once I started to emotionally heal that, it’s like, OK, no, the paperwork says I didn’t do anything wrong, I have to believe that like, I have to believe I didn’t do anything wrong, I just, unfortunately happened, he and I started to get better because it was something that emotionally broke me to be completely honest,…”* (ID:109, 27 y/o, non-Hispanic/non-Latina White)
During or Immediately After Loss Confirmed, Short time (Days to first few months), 14 to 31 Months After Loss Confirmed	Painful	Data was categorized as *painful* when participants indicated a general emotional distress from the loss.	*“Well, back in 2020, during COVID actually had two miscarriages back-to-back, one in July and one in October. The July one, I wasn’t that far along, I was like 5 weeks, so even though it was painful, uh, it didn’t upset me as much, but then after the next one, it upset me a lot because I was further along, I was like 11 weeks, almost 12, so that was very emotional for me.”* (ID: 102, 28 y/o, non-Hispanic/non-Latina Black)
During or Immediately After Loss Confirmed, Short time (Days to first few months), 14 to 31 Months After Loss Confirmed	Grief	Data was categorized as *grief* when participants indicated they were grieving or had deep sorrow over the loss of their baby.	*“…there were lots of tears, lots of, obviously sadness, grief, like a compounded grief almost, but it was harder, it was harder to figure out ways to process it because I didn’t have anything tangible.”* (ID: 57, 43 y/o, non-Hispanic/non-Latina White)
14 to 31 Months After Loss Confirmed	Unresolved Feelings	Data was categorized as *unresolved feelings* when participants indicated that they were still struggling with their grief, blame, and distress after 14 to 31 months since the loss.	*“I met, I still blame myself, which is silly, I still ask myself, like, did you accidentally take your migraine meds? Did you accidentally do this? Did you actually do? Did you eat something? But did you? And it’s, I know that’s silly, I know that’s just stupid and I wish, I wish I could stop that,…”* (ID: 63, 38 y/o, non-Hispanic/non-Latina White)
**Positive Emotions/Feelings**			
Before Loss Confirmed	Hope in the waiting	Data was categorized as *hope in the waiting* when participants described feeling a level of optimism about the pregnancy, even with the threat of miscarriage.	*“… I think because there is that, you know, hope and you do know that the embryo was put in there. There is, you know, a, I guess I don’t know, it’s probably not 50/50, but whatever, you know a chance that it will stick it’s a little bit more hopeful…”* (ID: 15, 37 y/o, Hispanic/Latina White)
**Thoughts**			
During or Immediately After Loss Confirmed	Denial	Data was categorized as *denial* when participants described not wanting to accept the reality of the miscarriage.	*“Well, I was hopeful that it was not, so I asked them to do another ultrasound because I didn’t want to accept that my baby was no more.”* (ID: 47, 30 y/o, Hispanic/Latina Other)
Short time (Days to first few months)	Uncontrolled Thoughts	Data was categorized as *uncontrolled thoughts* when participants described having thoughts they could not control as they waited to get into therapy.	*“I was having thoughts that I couldn’t control. Umm, you know, really like just I was just in a really bad space so to have to wait for months for therapy…”* (ID: 101, 33 y/o, non-Hispanic/non-Latina White)
Short time (Days to first few months)	Blame	Data was categorized as *blame* when participants assigned fault for the miscarriage to others or God.	*“I was also wanting to add that going through what I went through tested my faith. I was angry at God and questioned why he would give me a baby, just to take her away. And to go through it so many times. Miscarriage comes with so many complex emotions.”* (ID: 38, 33 y/o, non-Hispanic/non-Latina White)
During or Immediately After Loss Confirmed, Short time (Days to first few months)	Body Malfunction	Data was categorized as *body malfunction* when participants blamed their physiological body systems for not ‘doing what it was supposed to’, including holding the pregnancy/baby or recognizing the miscarriage, or when the initial miscarriage treatment was not successful and further miscarriage management was required.	*“…I just felt like, you know, my body wasn’t, it wasn’t accepting pregnancy at that time, I guess is the right wording to put, and then it at the same time it wouldn’t let go. So I was, you know, it was just really frustrating. Like, ok, well, I’ve already miscarried like, you know, it’d be great if my body would do what it’s supposed to like did the last time, but of course it didn’t.”* (ID: 30, 28 y/o, non-Hispanic/non-Latina White)
Short time (Days to first few months), 14 to 31 Months After Loss Confirmed	Comparing losses	Data was categorized as *comparing losses* when participants compared the loss(es) that they experienced during the COVID-19 pandemic to previous pregnancy losses they had or to what other women may be experiencing (minimizing their own current loss).	*“…I felt guilty for being so upset that I lost a baby that I never even knew, and there’s moms out there losing their kids to cancer because they actually know, and it made me feel so guilty for being so upset because like I, I didn’t even know this baby, it was tissue it was cells, it was, you know, like so that one that was a hard emotional thing to get over was telling myself that no, I shouldn’t feel guilty for still being upset, it was I wanted that baby.”* (ID: 109, 27 y/o, non-Hispanic/non-Latina White) *“Sometimes I feel guilty because I still cry because of what happened and well, I have a baby here, I mean, there are many women who don’t have babies and I have one and I’m crying, but…”* (ID: 47, 30 y/o, Hispanic/Latina Other)
During or Immediately After Loss Confirmed	Protecting Others	Data was categorized as *protecting others* when participants described wanting to shield others or not talk to others about their miscarriage to keep others from experiencing emotional distress.	*“And when I had the miscarriage, you know, when I started spotting on Halloween, I didn’t say anything to her [daughter] because I didn’t want to, you know, put that sort of stress on her, you know,…”* (ID: 19, 39 y/o, non-Hispanic/non-Latina White)
14 to 31 Months After Loss Confirmed	What Ifs	Data was categorized as *what ifs* when participants described wondering about the imagined life of the miscarried baby.	*“I think a lot about what it would be like to have those four kids in my house would be crazy, but also really wonderful and you know, I still think about them every day and about how old they would be and what they would be doing…”* (ID 57, 43 y/o, non-Hispanic/non-Latina White)
**Other Experiences**			
During or Immediately After Loss Confirmed, Short time (Days to first few months)	Compounding Effects	Data was categorized as *compounding effects* when participants described the accumulation of multiple stressors, including the COVID-19 pandemic on top of the miscarriage.	*“…like I really like, just couldn’t function. Like the anxiety, whatever, I mean, I think it was everything combined. I think the miscarriage in the middle of COVID on top of it being a third miscarriage on top of feeling, you know, like I had announced it and taking that from everybody else as well, not only me.”* (ID: 101, 33 y/o, non-Hispanic/non-Latina White) *“So a lot going on at that period of my life and you don’t really know how to grieve because you’re grieving so many things at one time.”* (ID: 72, 35 y/o, non-Hispanic/non-Latina White)
During or Immediately After Loss Confirmed	Dissociation/ Depersonalization	Data was categorized as *dissociation/ depersonalization* when participants described feeling disconnected from themselves (including thoughts/emotions), their bodies, and the world around them.	*“But at the time I wasn’t, you know, super….I mean, I was disappointed, but not, um, like it it, it didn’t feel like, I guess real, um, if that makes sense…”* (ID: 15, 37 y/o, Hispanic/Latina White) *“I don’t really know what happened in the hospital, I don’t know, I don’t know what happened, like I just, cause I didn’t have like ears like everything was just at home, you know, I didn’t know, so that’s and then I went home and I just was numb, I showered again, I was just numb for days,…”* (ID: 63, 38 y/o, non-Hispanic/non-Latina White)
During or Immediately After Loss Confirmed, Short time (Days to first few months), 14 to 31 Months After Loss Confirmed	Triggers	Data was categorized as *triggers* when a place, person, or situation reminded participants of their miscarriage and caused an emotional response.	*“We actually had a lady who went to our church too and our due dates were practically the same and she ended up having her baby on the due date of our second miscarriage, and I found myself, I hate saying not going to church because of that, but it was very hard, to sit behind her and see her with the baby carriage there for a little while.”*(ID: 30, 28 y/o, non-Hispanic/non-Latina White) *“I also think, you know, just from a miscarriage standpoint, like having to go into that office and sit down with all those other pregnant people, knowing that I’m not gonna look like them, right.*” (ID: 101, 33 y/o, non-Hispanic/non-Latina White)
14 to 31 Months After Loss Confirmed	Healing with Time	Data was categorized as *healing with time* when participants described lessening the intensity of negative emotions or thoughts about the miscarriage as time passed.	*“… I can think about the experience, I can talk about the experience and it doesn’t just like bring me to my knees every time, but it’s always there, I think there’s always an element of that loss and that grief that’s still a part of me and of my daily life, I don’t think about it every day anymore, it doesn’t feel as big in my life or in my every day, but I think it’s certainly still there and we’ll always be there.”* (ID: 104, 28 y/o, non-Hispanic/non-Latina Native Hawaiian or other Pacific Islander)

In Table 6, we arranged the categories along a chronological timeline of events to provide a cohesive view of participants’ experiences over time. This organization underscores the evolution and progression of participants’ experiences related to miscarriage within the context of the COVID-19 pandemic.

**Table 6:** Timeline of events and psychological emotions, feelings, thoughts, and experiences

Timeline of events	Emotions, feelings, thoughts, and experiences
Before loss confirmed	Anxiety (from other life events and previous pregnancy losses)
	Depression (from other life events)
	PTSD (from other life events and previous pregnancy losses)
	Hope in the waiting (to know if the current baby is fine)
	Trauma (from other life events and previous pregnancy losses)
	Guilt (from previous losses)
During or immediately after loss confirmed	Anxiety
	Lonely
	Trauma
	Helpless
	Grief
	Guilt
	Painful
	Triggers
	Denial
	Protecting Others
	Dissociation/ Depersonalization
	Isolated
	Shame
	Body’s Malfunction
	Compounding Effects
	Resentment
Short time (Days to first few months)	Anxiety
	Depression
	Helpless
	Anger
	Lonely
	Shame
	Isolated
	Triggers
	Resentment
	PTSD
	Body’s Malfunction
	Blame
	Uncontrolled Thoughts
	Compounding Effects
	Comparing Losses
	Guilt
	Grief
	Trauma
	Painful
14 to 31 Months after loss confirmed	Anxiety
	Depression
	PTSD
	Healing with Time
	What Ifs
	Comparing Losses
	Unresolved Feelings
	Grief
	Trauma
	Shame
	Resentment
	Guilt
	Painful
	Triggers

### Mental health disorders

The mental health disorder’s theme consists of three categories identified in our analysis including anxiety, depression, and PTSD. Participants reported being diagnosed with these conditions or exhibited symptoms suggesting these conditions. Prior to the pandemic miscarriage, some participants were already burdened with anxiety, depression, and PTSD, due to past life events including previous pregnancy losses. Anxiety was a prevalent condition, persisting through to the interviews, with women experiencing severe symptoms such as shaking and increased blood pressure, leading some to seek medication and therapy.

Depression and PTSD were not mentioned in conjunction with the occurrence of the miscarriage; however, they did re-surface shortly after the event and affected participants’ sleep, appetite, and mood, and led to flashbacks and panic attacks. As one participant described, “…*it’s literally like a flashback and you can feel like you’re actually in that same state, the same place where the trauma has like happened*.”

### Negative emotions/feelings

Participants shared a spectrum of negative emotions following their miscarriage, including anger at their situation, resentment towards others with successful pregnancies, and a profound sense of helplessness due to their inability to alter the outcome. These feelings were exacerbated by personal narratives of trauma, with some recounting the added distress of managing their miscarriage at home during the COVID-19 pandemic. This period not only intensified their current suffering but also revived past traumas, adding layers to their emotional distress. The emotional aftermath of miscarriage was characterized by deep loneliness and isolation, emotions that were heightened by the pandemic’s social distancing measures. Participants felt uniquely alone in their grief and their emotional distress was heightened by the perceived lack of understanding from others. The sentiment of isolation was reinforced when they were left physically alone during critical moments of loss, whether at home or in medical settings. This feeling of isolation was further exacerbated by the pandemic, due to strict adherence to quarantine and social distancing measures, underlining the unique challenges faced by individuals during such crises.

Shame and guilt emerged as significant emotional categories, with participants concealing their losses due to embarrassment or feelings of self-blame. These emotions persisted until the time of the interviews, indicating a long-term impact on mental health, with some women feeling responsible for their miscarriage. Lastly, the enduring pain, grief, and unresolved feelings encapsulate the long-term psychological toll of miscarriage. Participants described their experience as the most painful of their lives, with grief extending months beyond the loss. One participant described it as feeling like a “*gut punch*.” The absence of tangible elements to mourn in early miscarriages added complexity to their grieving process. For several participants, the intensity and duration of their sorrow led to psychiatric consultations, therapy, and medication in certain instances.

### Positive emotions/feelings

Amid their profound uncertainty, participants were found to have a single positive emotion: hope. This optimism was experienced as they awaited the definitive outcomes of their pregnancies, an anticipation that, unfortunately, ended in confirmed losses.

### Thoughts

Participants’ cognitive responses to miscarriage were categorized into categories such as denial, uncontrolled thoughts, blame, body malfunction, comparing losses, protecting others, and ‘what-ifs.’ Initially, some entered a state of denial, seeking reassurance through additional ultrasounds, despite an inner acknowledgment of the loss. For one woman, uncontrolled and intrusive thoughts were exacerbated due to lack of immediate access to therapy services. Blame was directed towards others or God, expressing feelings of anger towards God and perceiving their experiences as a test of their faith. Women also blamed their own bodies, perceived as failing to maintain the pregnancy, recognize the miscarriage, or respond to treatments, adding layers to their grief and questioning their physiological capability to sustain life.

Many women found themselves comparing their pandemic loss to previous pregnancy losses, amplifying their grief, especially when the loss occurred later in pregnancy or after hearing the baby’s heartbeat. The emotional distress was also intensified when the management of the miscarriage was unsuccessful, prolonging the physical and emotional process of loss. In one instance, a woman described how her provider was unable to give her mifepristone in the office to treat her incomplete miscarriage. Consequently, she had to travel several hours to a Planned Parenthood facility to obtain the medication, which ultimately proved ineffective, necessitating surgical intervention to remove the retained tissues.

Many participants also compared their pandemic loss to losses suffered by other women, often diminishing their own grief and fostering profound guilt for mourning a baby they never knew. Further, the burden of informing family members about the loss, coupled with the hesitation to announce subsequent pregnancies in efforts to protect others from experiencing these painful emotions, underscores the intricate emotional landscape navigated by these women. Additionally, the ‘what ifs’ category emerged when participants expressed thoughts about the life their miscarried child might have led, such as what it would have been like to have their child present in their day-to-day activities and imagining scenarios, such as birthday and holiday celebrations.

### Other experiences

Participants also reported experiences of compounding effects, triggers, dissociation/depersonalization, and healing with time. The “compounding effects” category was identified when participants expressed that the impact of their miscarriage was intensified by external factors, such as the stress from the ongoing pandemic (e.g., fear of contracting COVID-19, social distancing, job loss, caring for children and elderly, access to care), life stressors (e.g., moving to a new city, marital issues, financial issues), and reproductive stressors (e.g., multiple miscarriages, infertility, history of postpartum depression and/or anxiety).

Participants highlighted several triggers that evoked memories of their miscarriage, eliciting emotional responses. Some triggers induced reactions similar to PTSD symptoms (e.g., panic attacks), while other triggers provoked feelings of anger, sadness, shame, resentment, and grief. Triggers identified included seeing or hearing about other pregnant women, revisiting the clinic or ultrasound room where they received news of their loss, driving by the same hospital, and anniversary dates (e.g., due date, date loss confirmed).

The category termed “dissociation/depersonalization” captures participants’ experiences of emotional numbing and detachment from themselves, their bodies, and the world around them. Some described feeling detached from the reality of their miscarriage, accompanied by a blurred memory of when they learned of their loss, leading to a state of numbness where they shut out everything else. Despite all these challenges, there was a notable divergence in recovery paths; while some continued to struggle with grief and negative emotions even months after their loss, others reported healing over time and being in a much better place mentally at the time of the interviews.

### Integrated results

In Table 7, we present an integrated analysis of the qualitative and quantitative findings. The first column lists the common concepts of psychological experiences that were identified through either qualitative exploration or quantitative measurement. The second column displays the corresponding quantitative tool or a single item from the tool that was used to capture each psychological experience. The third column lists the qualitative category that the psychological experience aligns with. The last column denotes whether a convergence between the quantitative and qualitative outcomes was observed for each overarching psychological experience. Overall, data converged for 11 overarching psychological experiences, while four others were only captured qualitatively.

**Table 7:** Display of interpretive integration of quantitative and qualitative results

Overarching psychological experience	Quantitative measure	Qualitative category	Convergence
Trauma, Post-Traumatic Stress	PC-PTSD-5 RIMS Devastation Subscale	PTSD Trauma Dissociation/ depersonalization Triggers	Yes
Depression	PHQ-8	Depression	Yes
Anxiety	GAD-7	Anxiety Uncontrolled thoughts	Yes
Guilt	RIMS Isolation & Guilt Subscale (item #4) PC-PTSD-5 (item #5) PHQ-8 (item #6)	Guilt	Yes
Blame	PC-PTSD-5 (item #5)	Blame	Yes
Body’s malfunction	RIMS Isolation & Guilt Subscale (item # 3)	Body’s malfunction	Yes
Lonely & Isolated	RIMS Isolation & Guilt Subscale	Lonely Isolated	Yes
Stress	PSS-4	Compounding effects	Yes
Grief & Loss	RIMS Loss of Baby Subscale	What ifs Grief	Yes
Loss of control	RIMS Devastation Subscale (item # 2) PSS-4 (item #1)	Helpless	Yes
Shame	RIMS Isolation & Guilt Subscale (item #5)	Shame	Yes
Negative emotions/feelings		Unresolved feelings Anger Resentment Painful	No
Positive emotions/feelings		Hope in the waiting	No
Thoughts		Comparing losses Denial Protecting others	No
Other experiences		Healing with time Triggers	No

## Discussion

The psychological consequences of miscarriage, exacerbated by the COVID-19 pandemic’s stressors, are a central focus of this study, aiming to explore the psychological experiences, quantify the levels of psychological distress (depression, anxiety, and/or PTSD), and examine the relationships between personal significance of miscarriage, perceived stress, and psychological distress of women in North Carolina who suffered a miscarriage of a desired pregnancy between March 30, 2020, and February 24, 2021, of the COVID-19 pandemic, at 14 to 31 months after the loss.

Findings revealed that 47.9% and 38.0% of the sample reported moderate to severe anxiety and depression respectively, 14 to 31 months after their miscarriage. Strikingly, the likelihood of PTSD related to pandemic miscarriage was observed in 64.8% of the sample. Compared to studies conducted prior to the COVID-19 pandemic, our findings suggest that the prevalence of post-miscarriage psychological distress may have been heightened during the time of the pandemic and the effects persisted up to 31 months after. For example, Farren et al. [9], reported that nine months after a miscarriage, 18% of women met the criteria for post-traumatic stress, 17% reported moderate/severe anxiety, and 6% reported moderate/severe depression. Similarly, Volgsten et al. [39] found that depression symptoms decreased over time and symptoms observed at four months post-miscarriage did not differ from women without miscarriage. The elevated psychological distress levels in our sample align with our observed average COVID-19 stress rating (3.76 out of 5) and suggest moderate to high levels of pandemic-related stress. Our qualitative results further underscore the compounded nature of stressors that these women faced during the pandemic, which might have amplified the emotional distress experienced post-miscarriage.

Our study further identified key predictors that influence the likelihood of women meeting the clinical cut-off for depression, anxiety, and PTSD following pandemic miscarriage. We identified a significant positive association between perceived stress post-miscarriage and both depression and anxiety. These findings are in some ways consistent with those of Chen et al. [40], who also highlighted the role of perceived stress as a predictor for depressive symptoms, albeit in a broader population of women of childbearing age. Our qualitative findings further elaborate on this within the context of miscarriage, as participants described anxiety, depression, and PTSD symptoms, as well as triggers, uncontrolled thoughts, and experiences of dissociation and depersonalization. These findings highlight the need for healthcare providers to deliver targeted empathetic support to mitigate mental health symptoms post-miscarriage. It is essential to implement and assess strategies, such as separate waiting areas in OB/GYN clinics and rainbow clinics [41] (a clinic where specialists care for patients, specifically, those who have experienced perinatal loss), in alleviating the stress and triggers of women who have experienced perinatal loss.

The associations we found between prior trauma and increased risk for depression and PTSD post-miscarriage are consistent with previous literature indicating that pre-existing traumatic experiences may predispose individuals to exacerbated psychological responses following subsequent distressing events [42]. For example, individuals who experienced adverse childhood experiences are more likely to exhibit PTSD symptoms in adulthood compared to individuals without such experiences [43]. Similarly, women previously exposed to intimate partner violence are more likely to experience anxiety, depression, PTSD, and other mental health disorders [44]. Given these findings, it is crucial for women with a history of trauma, particularly when coupled with reproductive trauma like miscarriage, to receive further psychological assessments and appropriate referrals to address their mental health needs.

The protective influence of subsequent childbirth on all mental health outcomes in our study is a noteworthy finding. Previous studies suggest that while subsequent pregnancies can evoke anxiety due to fear of repeated loss [45], a successful subsequent pregnancy can offer emotional healing and reduce symptoms of grief and trauma [46]. Our study results align with these findings. During interviews, women in the subsample were hesitant to share news of their subsequent pregnancies due to fear of another loss, however, some also spoke of healing with time and quantitatively we determined that having a child helped them lessen the intensity of their distress towards their miscarriage.

An alternative explanation regarding the lower mental health symptoms observed in those who were pregnant/had conceived since the miscarriage compared to those who were not pregnant/had not conceived is the role of intentionality in conceiving post-miscarriage. It is possible that those who were experiencing greater psychological distress were less inclined to attempt conception, either due to a sense of not being ready or fear of another loss. This could imply that the decision to try for another pregnancy may itself be influenced by a woman’s emotional state post-miscarriage. We did not collect this data; however, future studies should investigate this correlation.

Women in our study appraised their miscarriage as a devastating event, feeling isolated, guilty, and experiencing profound grief over the loss of their baby. The higher the personal significance they attributed to the miscarriage, the higher the likelihood of PTSD- by a factor of 1.12 times at 14 to 31 months post-miscarriage. Volgsten et al. [39] found that there was no change in personal significance from 1-week to 4-weeks post-miscarriage, indicating that the personal significance attributed to a miscarriage remains consistent over that period. However, participants in their study reported lower RIMS scores compared to ours, suggesting that women who experienced miscarriage during the pandemic may attribute greater personal significance to their loss compared to those before the pandemic. During our interviews, women often described feeling guilt, shame, and helplessness in relation to their miscarriage. They also felt a sense of betrayal from their bodies, sentiments that closely align with items from RIMS. Additionally, women compared their loss to those of other women or to their own prior losses, likely as an attempt to reappraise the personal significance of their miscarriage to lessen their negative emotions and feelings about their miscarriage.

Additionally, many women in our study described thinking about their miscarried baby often and daydreaming about the life the baby would have lived. Deliberate rumination can be helpful in processing traumatic experiences toward positive post-traumatic growth, or a positive psychological change, after miscarriage [47], however intrusive rumination can inhibit post-traumatic growth [48]. Thus, it is vital for health care and mental health professionals to discern the type of rumination taking place and its impact on the individual’s healing. It is unclear if women in our study found thinking about the “What Ifs” of their baby was helpful or harmful in their healing journey and this warrants further exploration.

Further, the combination of grieving a miscarriage while navigating the compounding stresses, isolation, and uncertainties of a global pandemic likely created a uniquely challenging environment for affected women. Throughout the interviews, women frequently highlighted various compounding stressors that intensified their psychological distress. These ranged from personal life challenges such as relocating or job transitions to pandemic-specific stressors experienced during mandated stay-at-home orders. These findings align with Heaney and Galeotti’s [49] study of women in Ireland who suffered a miscarriage during the COVID-19 pandemic. In their research, women similarly reported heightened feelings of isolation, loneliness, and anxiety. Given these converging findings, it becomes imperative for healthcare providers, mental health professionals, and policymakers to recognize the heightened psychological distress faced by women experiencing miscarriages during global crises.

Furthermore, although only one participant noted that the changing legislation surrounding Dobbs v. Jackson Women’s Health Organization impacted her ability to receive a prescription for mifepristone from her provider, this significant issue was affecting women’s health rights nationwide during our data collection period [50]. A lawsuit targeting the US Food and Drug Administration’s approval of mifepristone, used in medication abortions and to treat miscarriages, created widespread confusion among healthcare providers leading to leading to more cautious or even refusal to provide standard miscarriage management [50]. This hesitancy raises concerns about prolonged physical and emotional distress for patients, as providers navigate the fear of potential legal repercussions. These developments underscore the need to examine the broader implications of such legal threats on miscarriage management and patient well-being.

Lastly, it’s apparent that while some qualitative results did not converge with our quantitative data, this divergence actually highlights significant gaps in the current measurement tools, which fail to capture a range of emotions such as anger, resentment, and healing over time. Nonetheless, these wide ranging emotions are consistent findings among prior studies [51–53]. They add depth to our understanding of the psychological impacts and emphasize the emotional and cognitive complexity of miscarriage within the context of a global pandemic. Future research could focus on refining psychometric tools to include these uncovered aspects, ensuring a comprehensive evaluation of the psychological impacts of miscarriages.

### Limitations

This study has several limitations, including its cross-sectional design, which limits the ability to determine causation. Additionally, it lacks data on women’s stress, PTSD, anxiety, or depression levels before and immediately after miscarriage, affecting the understanding of distress over time. Not using the RIMS tool to measure the personal significance of miscarriage near the time of the event introduces potential recall or retrospective bias. Additionally, the lower reliability of the PC-PTSD-5 tool might reflect sample homogeneity rather than measurement accuracy. The study met its estimated sample size, yet some broad confidence intervals in the logistic regression analyses suggest caution in interpreting the odds ratios due to potential imprecision. Recruitment methods, primarily online via Facebook, may introduce selection bias, and retrospective accounts of miscarriage experiences could lead to recall bias. Efforts to include racially and ethnically diverse participants, including Spanish speakers, were made, yet most women did not identify as racial or ethnically diverse individuals.

### Clinical significance/implications

It’s essential for healthcare providers to conduct thorough assessments, screening for mental health histories and prior traumas to identify those at risk for post-miscarriage mental health issues. Although no single tool for assessing mental health post-miscarriage exists, our study recommends using a range of validated tools (e.g., GAD-7, PHQ-8, PSS-4) in clinical settings, not just in the period immediately following a miscarriage, but during post-miscarriage follow-up visits and wellness annual evaluations throughout a woman’s lifespan.

An interdisciplinary approach, incorporating psychologists, social workers, and specialists, is crucial for holistic care. If mental health issues are identified or suspected, follow-up appointments and immediate referral to mental health services should be made. Healthcare agencies should also facilitate support groups to alleviate loneliness and isolation by allowing women to share their experiences [54], particularly during another mandated stay-at-home order.

Healthcare providers should educate patients about the normal range of emotions after miscarriage and clear indicators for when to seek further mental health care. Referrals to mental health specialists may be necessary when emotional symptoms persist, cause substantial distress, or interfere with daily functioning [55]. By providing educational resources to women and their families about miscarriage and offering emotional support, healthcare professionals can validate these feelings. Encouraging open discussions about the experience can help to normalize the event, facilitate healing, and potentially reduce feelings of isolation and loneliness [56].

Furthermore, providers should adopt a trauma-informed care approach in their practice. Trauma-informed care aims to avoid re-traumatization by understanding a patient’s history of trauma and integrating trauma-sensitive procedures into their care protocols (e.g., having a separate waiting area for those who recently had a pregnancy loss). All staff should be educated to provide a secure emotional environment where patients feel comfortable discussing concerns without judgment, foster open and honest communication, and incorporate trauma-sensitive interviewing [12]. Healthcare providers should include coping techniques as part of the post-miscarriage care plans, ranging from mindfulness exercises to managing distressing triggers [57]. It is important that providers recognize the uniqueness of each person’s experience and tailor care plans accordingly to provide patient-centered, trauma-informed care. Additionally, healthcare systems should be prepared for future pandemics, epidemics, or other crises by developing crisis-specific resources, interventions, and guidelines. These may include evidence-based guidelines on delivering sensitive news remotely and efficient telehealth support services tailored to individual patient needs [49].

### Future research

This study lays the groundwork for future research to develop interventions that address psychological distress following a miscarriage, especially during times of heightened stress and social isolation. Future research could consider using Lazarus & Folkman’s [58] theory of transactional stress and coping as a framework to explore coping strategies in women who experience miscarriage. This theory has been used as a theoretical framework in other studies exploring miscarriage [59, 60] and could provide additional insight into the experience of miscarriage during the COVID-19 pandemic. Randomized controlled trials could be useful in determining the effectiveness of various intervention strategies, including online support groups, trauma-informed care clinics, rainbow clinics, cognitive-behavioral therapies, and other community-based resources (e.g., bereavement care packages and resilience training). Moreover, limited prior research indicates that partners also suffer greatly post-miscarriage [7]. Further research is warranted to explore the impact of miscarriage on partners’ mental well-being and the dynamics within couples, especially considering the added stressors of a global pandemic or other crises. Additionally, this study calls for more diverse samples and long-term research to understand the psychological aftermath of miscarriage fully and to ensure the relevance and effectiveness of support across different communities.

## Conclusions

This study sought to understand the compounded psychological impact of experiencing a miscarriage during the unprecedented circumstances of the COVID-19 pandemic. Our findings indicate that the pandemic not only heightened the overall prevalence of psychological distress, such as depression, anxiety, and PTSD among the affected women but also added layers of complexity to their emotional experiences, in some cases extending up to 31 months post-miscarriage. The data revealed key predictors like prior trauma and perceived stress, as well as protective factors like subsequent childbirth, which influence the psychological well-being of women after miscarriage. The study underscores the need for comprehensive mental health screenings, specialized support for vulnerable groups, and the necessity of trauma-informed care. Providers are strongly encouraged to adopt a multifaceted, individualized approach to patient care that is cognizant of the unique stressors introduced by the pandemic.

### Electronic supplementary material

Below is the link to the electronic supplementary material.

Supplementary Material 1

Supplementary Material 2

## Data Availability

The datasets used and/or analyzed during the current study are available from the corresponding author on reasonable request.
