# Supplementary material for: Compounding stress: A mixed-methods study on the psychological experience of miscarriage amid the COVID-19 pandemic
Source: BMC Pregnancy Childbirth. 2024 Jun 13;24:426. doi: 10.1186/s12884-024-06610-z (PMC11170813; doi:10.1186/s12884-024-06610-z)
Supplement: Supplementary file 1 — Supplementary Material 1 [file 12884_2024_6610_MOESM1_ESM.docx]

Table A

*Revised Impact of Marriage Scale Items (N = 71)*

Scale Definitely/quite true

Item n %

Isolated/guilt

1. I feel much alone in my loss 60 85

2. My miscarriage destroyed my zest for life 28 39

3. I feel my body has betrayed me 54 76

4. I feel guilty about my miscarriage 44 62

5. Through my miscarriage, I have experienced a loss of pride in myself 36 51

6. I feel very isolated by my miscarriage 45 63

Loss of baby

1. Through miscarriage, I feel I lost a part of myself 56 79

2. I feel there will always be a place in my heart for the miscarried baby 68 96

3. Through my miscarriage, I feel that I have lost a person 57 80

4. I get irritated when my miscarried baby is called a fetus 44 62

5. I dwell on the fact my miscarried child will only exist in my memory 50 70

Devastating event

1. My miscarriage was a horrendous, devastating event 61 86

2. Miscarriage equals one big loss of control 58 82

3. Miscarriage is like going from happiness to total unhappiness 62 87

4. Miscarriage is a nightmare 64 90

5. My miscarriage represents a major setback for me 52 73
